# Morphometric Evaluation of Korean Femurs by Geometric Computation: Comparisons of the Sex and the Population

**DOI:** 10.1155/2015/730538

**Published:** 2015-08-27

**Authors:** Ho-Jung Cho, Dai-Soon Kwak, In-Beom Kim

**Affiliations:** Department of Anatomy, Catholic Institute for Applied Anatomy, The Catholic University of Korea, 222 Banpo-Daero, Seocho-gu, Seoul 137-701, Republic of Korea

## Abstract

We measured 28 parameters of 202 femurs from Koreans by an automated geometric computation program using 3D models generated from computed tomography images. The measurement parameters were selected with reference to physical and forensic anthropology studies as well as orthopedic implant design studies. All measurements were calculated using 3D reconstructions on a computer using scientific computation language. We also analyzed sex and population differences by comparison with data from previous studies. Most parameters were larger in males than in females. The height, head diameter, head center offset, and chord length of the diaphysis, most parameters in the distal femur, and the isthmic width of the medullary canal were smaller in Koreans than in other populations. However, the neck-shaft angle, subtense, and width of the intercondylar notch in the distal femur were larger than those in other populations. The results of this study will be useful as a reference for physical and forensic anthropology as well as the design of medical devices suitable for Koreans.

## 1. Introduction

The femur is the largest bone in the human body. Its proximal part and the pelvis constitute the hip joint, and its distal part constitutes part of the knee joint. Therefore, the femur is widely researched in fields such as physical and forensic anthropology, human kinematics, and orthopedics. Physical and forensic anthropology research involves using metric or nonmetric methods to determine differences in the femur with respect to populations, sex, and age [1–10]. In addition, orthopedics research involves analysis of the femoral head, neck, and the proximal part of the medullary canal for hip joint studies [11–33] as well as the shape of the distal part of the femur for knee joint studies [34–47]. Furthermore, some studies have investigated the shape of the medullary canal and femoral curvature to design intramedullary fixators and investigated the axes for orthopedic surgery [23, 48–51].

Most of those studies used bones from cadavers or patients who underwent surgery. Moreover, efforts have been made to reduce inter- and intraobserver measurement errors when using dry bone, radiography, and 3D models. Although some studies have used digital methods [21, 30, 52], they have focused only on portions of the femur.

Therefore, this study morphometrically evaluated 28 parameters of 202 femurs from Koreans by an automated geometric computation program using 3D models generated from computed tomography (CT) images. Furthermore, we calculated the size of the medullary canal for implant stem and intramedullary device design. Finally, we analyzed sex and population differences by comparison with data from previous studies.

## 2. Materials and Methods

The study included 202 femurs from Koreans from the Catholic Digital Human Library (November 2003 to present), which was established from CT images from the whole bodies of cadavers. CT images had a slice thickness of 0.75 or 1.0 mm and a pixel dimension from 0.431 to 0.832 mm. CT scans of cadavers alongside a plastic ball of known size (diameter: 2.25 inches) for calibration were used to construct 3D skeleton models. The images obtained were reconstructed in 3D skeleton models created by a 3D reconstruction program (Mimics, version 16, Materialise, Belgium). The gray-level threshold value at the time of the 3D reconstruction was determined by comparing the actual and three-dimensional volumes of the plastic ball. Thus, the size of the 3D reconstructed bone models was not different from that of the real bones (*P* = 0.74).

We selected femurs with no congenital anomalies or pathological deformities as determined by a radiologist and anatomist. Demographic information including sex, age, and height was available for each sample. The mean age and height of male samples (*n* = 88) were 50 years and 167 cm, respectively; those of female samples (*n* = 114) were 54 years and 156.4 cm, respectively. We examined 102 and 100 left and right femurs, respectively. All measurements and calculations were conducted using 3D reconstructions on a computer using Matlab (version R2011, MathWorks, MA, USA).

**Figure 1:**
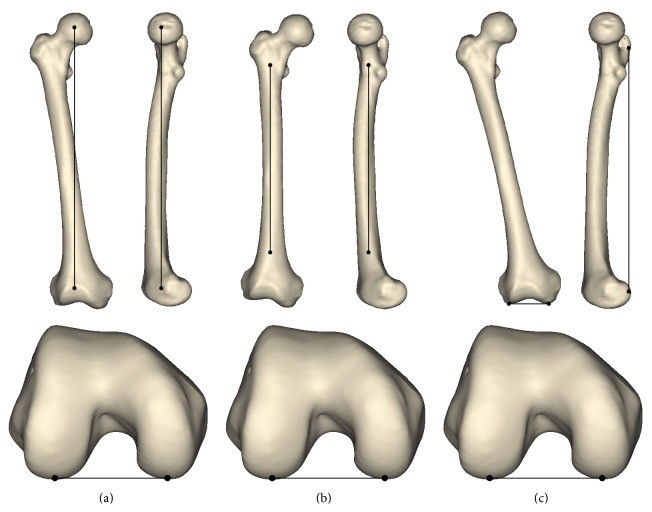
Three alignment methods. (a) Mechanical axis alignment; (b) anatomical axis alignment; (c) alignment by osteometric board.

Prior to measurement, we aligned the femurs by using 3 different methods. In the first method, we aligned the mechanical axis of the femur in the sagittal and coronal planes as described by Seo et al. [53]; the mechanical axis was defined as the line connecting the center of the femoral head to the apex of the intercondylar notch (Figure 1(a)). In the second method, we aligned the anatomical axis of the femur in the sagittal and coronal planes; the anatomical axis was defined as a least-square-fitting line calculated from the diaphysis (Figure 1(b)). The third method was osteometric board alignment. The most inferior points of both condyles were aligned in a transverse plane. In all methods, the extreme posterior points of the medial and lateral condyles were aligned in the coronal plane (Figure 1(c)).

After the alignment procedure, we exported the 3D femur models to stereolithography format file for geometric computation. The geometric computation software, which was programed in Matlab, had 3 basic functions. The first function was finding extreme points: the most superior, inferior, anterior, and posterior points were located, and the distances between them were calculated. The second function was least square primitive geometric fitting by line, sphere, and cylinder; the anatomical and mechanical axes of the femur were located, and the angle between them was calculated. The third function was section reconstruction, in which arbitrary sectional images of 3D objects were created and used to calculate sectional parameters. Our in-house coding program was verified using simple solid primitives (i.e., a sphere, hexahedron, and cylinder). Then, we randomly chose 10 samples to compare the measurement results of our program with those of commercial stereo lithography computer-aided design software (3-matic version 8.0, Materialise, Belgium). There were no differences in any parameter between programs (*P* = 0.71).

**Figure 2:**
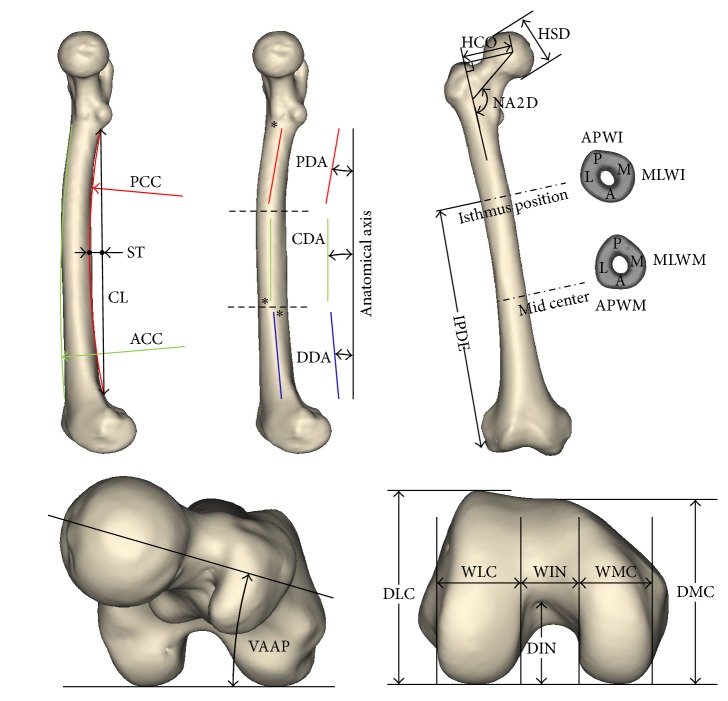
Measurement parameters (see Table 1 for definitions).

Measurement parameters were selected with reference to physical and forensic anthropology studies as well as orthopedic implant design studies. A total of 28 variables were measured by using the models: the whole femur, including the length and axes of the whole femur; proximal femur, including the sizes and angles of the head and neck; diaphysis, including the length, curvature, and angle of the femoral shaft; distal femur, including the sizes of the condyle and intercondylar notch; and medullary part, including the isthmic position and the size of the cross sections of the medullary canal (Table 1 and Figure 2).

Data were analyzed using SPSS (version 17.0; SPSS Inc., Chicago, IL, USA). Independent *t*-tests were performed to assess differences in the means of variables between sexes and population by comparison with data from previous studies after the data were tested for normality of distribution by the Kolmogorov-Smirnov test. Variables that did not exhibit a normal distribution were analyzed by the Mann-Whitney *U*test. The level of significance was set at *P* < 0.05.

## 3. Results and Discussion

### 3.1. Comparison of Femur Parameters between Sexes

The results of the automatic geometric calculations are shown in Table 2. The height of the whole femur was calculated after applying the 3 alignment methods mentioned above. The greatest height was aligned by using the mechanical axis (HMA, 417.41 ± 23.73 mm), followed by the anatomical axis (HAA, 416.96 ± 23.90 mm) and the osteometric board (HOB, 414.52 ± 23.66 mm). There were statistical differences among alignment methods (*P* < 0.01), and all results were significantly greater in male samples than in female samples (*P* < 0.01). The mean angle between mechanical and anatomical axes in the coronal plane (AMAC) did not differ statistically between sexes (*P* = 0.57). In the sagittal plane, the angle between mechanical and anatomical axes (AMAS) in females (3.80 ± 0.88°) was more posteriorly inclined than that in males (3.07 ± 1.03°) (*P* < 0.01).

At the proximal part of the femur, the femoral head diameter (HSD) was significantly greater in males (48.50 ± 2.23 mm) than in females (43.25 ± 2.12 mm) (*P* < 0.01). The femoral head center offset (HCO), which is the distance between the femoral head center and anatomical axis, was not statistically different between sexes (*P* = 0.72). The femoral neck-shaft angle in the coronal plane as projected in 2D and 3D planes (NA2D and NA3D, resp.) was not statistically different between sexes (*P* = 0.18 and *P* = 0.60, resp.). However, there were statistical differences between the 2D and 3D angles (*P* < 0.01). The version angle on axial plane (VAAP) was significantly greater in females (20.34 ± 10.54°) than in males (14.61 ± 10.30°) (*P* < 0.01); also, 94.74% of samples exhibited anteroversion.

We divided the diaphysis into 3 equal parts. The angles between the proximal, central, and distal diaphysis and the anatomical axis in the sagittal plane (PDA, CDA, and DDA, resp.) were 7.56 ± 1.55°, −0.23 ± 0.52°, and −7.08 ± 1.71°, respectively. The angles of the proximal and central parts of the diaphysis differed statistically between sexes (both *P* < 0.01). The anterior cortex curvature in the sagittal plane (ACC) tended to be greater in males (1381.04 ± 473.02 mm) than in females (1350.98 ± 741.17 mm), although the difference was not significant (*P* = 0.06). The posterior cortex curvature in the sagittal plane (PCC) was significantly greater in males (889.69 ± 188.15 mm) than in females (755.65 ± 160.78 mm) (*P* < 0.01). The chord length (CL) of the diaphysis was statistically longer in males (289.61 ± 16.56 mm) than in females (274.47 ± 14.95 mm) (*P* < 0.01). There was no statistic difference in subtense (ST) length between females and males (*P* = 0.35).

At the distal part of the femur, the depth and width of the lateral condyle (DLC and WLC, resp.), depth and width of the medial condyle (DMC and WMC, resp.), and depth and width of the intercondylar notch (DIN and WIN, resp.) were statistically smaller in females than in males (all *P* < 0.01).

Regarding the medullary canal, the isthmic position of the medullary canal (IPDE) from the distal end of the femur was significantly greater in males (434.19 ± 23.90 mm) than in females (403.97 ± 18.10 mm) (*P* < 0.01). The mediolateral and anteroposterior widths of the isthmus (MLWI and APWI, resp.) did not differ statistically between sexes (*P* = 0.98 and *P* = 0.22, resp.). In the medullary canal at the mid center of the femoral shaft, the mediolateral anteroposterior widths (MLWM and APWM, resp.) were statistically larger in males than in females (11.48 ± 1.96 versus 10.24 ± 1.82 mm and 13.71 ± 2.19 versus 12.41 ± 2.08 mm, resp.; both *P* < 0.01).

### 3.2. Comparison of Femur Parameters between Koreans and Other Populations

We compared the results of the present study with those of previous studies using adult femurs. We also compared sex differences between the present and previous studies that contained relevant data. The parameters of the whole and proximal femur by population are shown in Table 3. The height of the whole femur was measured by 3 methods. It should be noted that there were statistic differences among all 3 methods (*P* < 0.01). Whole femur height measured by HOB in Koreans was significantly shorter than that in African American populations I and II, British, European Americans, male North American Indians I, and North American Indians II (all *P* < 0.05), but not statistically different from that in Inuit and female North American Indians I [2, 9]. We were unable to compare our data with those of Americans, French, or Germans, because the measurement axis was unspecified or different from that used in the present study [22, 23, 28, 36].

Regarding the proximal part of the femur, the HSD of Koreans was not statistically different from those of the French, Turks, or Americans [11, 22, 23]. However, the HSD of Pakistanis and the French was statistically larger and smaller than that of Koreans (both *P* < 0.01) [28, 31]. HCO was statistically smaller in Koreans than in the Swiss French, the French, Pakistanis, Turks, and Americans (all *P* < 0.01) [11, 22, 23, 27, 28, 31]. NA2D was statistically larger in Koreans than in the French, Americans, and Turks (all *P* < 0.01) [11, 22, 23, 28]; however, there was no difference compared to that of the Swiss French or Pakistanis [27, 31]. At the proximal part of the femur, the HSD of Koreans was similar to that of other populations. However HCO was smaller and NA2D was larger in Koreans compared to those of other populations (Table 3).

At the diaphysis (Table 4), CL was statistically longer in Koreans than in Japanese populations II and III (*P* < 0.05) [51]. CL was statistically shorter in Koreans than African American populations I and II, European American populations I and II, North American Indian populations I–III, Inuit, and Japanese population I (all *P* < 0.01) [9, 51]. ST was statistically larger in Koreans than in all other populations (*P* < 0.01), except that of male North American Indian population I [9]. This indicates Korean femurs generally have a greater sagittal curve. In most previous studies, ST was larger in males than in females. However, ST was larger in females in the Korean population, African American population II [9], and Japanese population III than in males [51]. In addition, there was no sex difference in ST in North American Indian population III [51].

At the distal part of the femur (Table 5), DLC was statistically smaller in Koreans than in Germans in both sexes (both *P* < 0.01) [36]. WLC was statistically smaller in Koreans than in Germans in both sexes (both *P* < 0.01) [36]. Moreover, WLC tended to be larger than that in the Japanese and the Taiwanese, although not statistically [38, 40]. DMC was statistically smaller in Koreans than in Germans in both sexes (both *P* < 0.01) [36]. WMC was statistically smaller in Koreans than in Germans, the Japanese, and the Taiwanese (all *P* < 0.01) [36, 38, 40]. DIN was statistically smaller in Koreans than in Germans in both sexes (both *P* < 0.01) [36]. WIN was statistically larger in Koreans than in Germans and the Taiwanese (both *P* < 0.01), except for German females [36, 38]. Thus, the WLC of Koreans was similar to that of most other populations except Germans, and WIN was larger than that in other populations; meanwhile, all other parameters in the distal femur were smaller in Koreans.

At the medullary canal (Table 5), both MLWI and APWI were statistically smaller in Koreans than in Turks and Americans (both *P* < 0.01) [11, 23].

## 4. Conclusion

We calculated the 28 morphometric parameters of femurs from Koreans by using a geometric computation program. The results show that most parameters were larger in males than in females. Moreover, 14 variables differed statistically between Koreans and other populations.

These data can be used for studies in physical and forensic anthropology as well as orthopedic implant design. Many previous studies only measured specific regions of the femur, such as the proximal and distal parts for the hip and knee joints, respectively. However, data of the whole femur are more useful for the aforementioned purposes. Traditional direct measurement methods require many times whole femur study. On the other hand, automated software can rapidly analyze the whole femur as well as other bones. Also, automated computation methods have lower inter- and intraobserver variations than traditional direct measurement methods.

We expect that the Korean data and comparisons with other populations will be useful references for physical and forensic anthropology as well as orthopedic device design. In addition, this computational measurement method may be useful for surgical navigation systems.

## Figures and Tables

**Table 1:** Definitions of femur parameters.

Group	Abbreviation	Measurement
Whole	HMA	Height after mechanical axis alignment
	HAA	Height after anatomical axis alignment
	HOB	Height measured by osteometric board
	AMAC	Angle between mechanical axis and anatomical axis in coronal plane
	AMAS	Angle between mechanical axis and anatomical axis in sagittal plane

Proximal	HSD	Sphere diameter fit to head
	HCO	Head center offset
	NA2D	Neck angle on coronal plane
	NA3D	Neck angle in 3D vector
	VAAP	Version angle on axial plane

Diaphysis	PDA	Proximal diaphysis (1/3) angle on sagittal plane
	CDA	Central diaphysis (2/3) angle on sagittal plane
	DDA	Distal diaphysis (3/3) angle on sagittal plane
	ACC	Anterior cortex curvature on sagittal plane
	PCC	Posterior cortex curvature on sagittal plane
	CL	Chord length
	ST	Subtense

Distal	DLC	Depth of lateral condyle
	WLC	Width of lateral condyle
	DMC	Depth of medial condyle
	WMC	Width of medial condyle
	DIN	Depth of intercondylar notch
	WIN	Width of intercondylar notch

Medullary canal	IPDE	Isthmic position from distal end
	MLWI	Mediolateral width at isthmus
	APWI	Anteroposterior width at isthmus
	MLWM	Mediolateral width at mid center
	APWM	Anteroposterior width at mid center

**Table 2:** Comparison of femur parameters by sex.

Group	Abbreviation	Female		Male		Combined		*P*
		Mean	SD	Mean	SD	Mean	SD	

Whole	HMA (mm)	404.65	18.11	434.43	19.29	417.41	23.73	<0.01
	HAA (mm)	403.97	18.10	434.19	23.90	416.96	23.90	<0.01
	HOB (mm)	401.71	17.87	431.62	23.66	414.52	23.66	<0.01
	AMAC (deg.)	5.49	0.89	5.42	0.82	5.46	0.86	0.57
	AMAS (deg.)	3.80	0.88	3.07	1.03	3.49	1.02	<0.01

Proximal	HSD (mm)	43.25	2.12	48.50	2.23	45.50	3.39	<0.01
	HCO (mm)	37.26	5.40	38.69	5.29	37.88	5.39	0.72
	NA2D (deg.)	130.80	6.34	129.56	6.09	130.27	6.25	0.18
	NA3D (deg.)	127.70	5.80	128.16	6.35	127.90	6.06	0.60
	VAAP (deg.)	20.34	10.54	14.61	10.30	17.89	10.79	<0.01

Diaphysis	PDA (deg.)	7.31	1.60	7.90	1.42	7.56	1.55	<0.01
	CDA (deg.)	−0.08	0.47	−0.43	0.52	−0.23	0.52	<0.01
	DDA (deg.)	−7.22	1.85	−6.89	1.49	−7.08	1.71	0.18
	ACC (mm)	1350.98	741.17	1381.04	473.02	1364.16	636.25	0.06^*∗*^
	PCC (mm)	755.65	160.78	889.69	188.15	814.43	185.25	<0.01
	CL (mm)	274.47	14.95	289.61	16.56	281.11	17.35	<0.01
	ST (mm)	13.82	2.83	13.43	2.87	13.65	2.85	0.35

Distal	DLC (mm)	58.39	2.76	64.63	3.65	60.11	4.43	<0.01
	WLC (mm)	24.05	2.00	27.96	1.91	25.76	2.76	<0.01
	DMC (mm)	55.25	3.02	61.22	3.06	57.85	4.24	<0.01
	WMC (mm)	23.46	2.39	25.78	1.85	24.47	2.45	<0.01
	DIN (mm)	27.16	1.85	30.50	2.05	28.61	2.25	<0.01
	WIN (mm)	18.97	2.75	21.66	2.66	20.14	3.02	<0.01

Medullary canal	IPDE (mm)	403.97	18.10	434.19	23.90	416.96	23.90	<0.01
	MLWI (mm)	9.59	1.93	9.60	1.94	9.60	1.93	0.98
	APWI (mm)	10.97	2.60	11.51	2.35	11.24	2.49	0.22
	MLWM (mm)	10.24	1.82	11.48	1.96	10.77	1.97	<0.01
	APWM (mm)	12.41	2.08	13.71	2.19	12.97	2.22	<0.01

^*∗*^The result of nonparametric test by Mann-Whitney *U* test.

HMA: height after mechanical axis alignment, HAA: height after anatomical axis alignment, HOB: height measured by osteometric board, AMAC: angle between mechanical axis and anatomical axis in coronal plane, AMAS: angle between mechanical axis and anatomical axis in sagittal plane, HSD: sphere diameter fit to head, HCO: head center offset, NA2D: neck angle on coronal plane, NA3D: neck angle in 3D vector, VAAP: version angle on axial plane, PDA: proximal diaphysis (1/3) angle on sagittal plane, CDA: central diaphysis (2/3) angle on sagittal plane, DDA: distal diaphysis (3/3) angle on sagittal plane, ACC: anterior cortex curvature on sagittal plane, PCC: posterior cortex curvature on sagittal plane, CL: chord length, ST: subtense, DLC: depth of lateral condyle, WLC: width of lateral condyle, DMC: depth of medial condyle, WMC: width of medial condyle, DIN: depth of intercondylar notch, WIN: width of intercondylar notch, IPDE: isthmic position from distal end, MLWI: mediolateral width at isthmus, APWI: anteroposterior width at isthmus, MLWM: mediolateral width at mid center, and APWM: anteroposterior width at mid center.

**Table 3:** Comparison of parameters of the whole and proximal femur among populations.

Measurement		Population	Female	Male	Combined
Group	Abbreviation				
Whole	HOB (mm)	Korean (this study)	401.71	431.62	414.52
		Inuit [9]	405.6	430	
		North American Indian I [9]	399.3	445.2	
		North American Indian II [9]	419.2	443.4	
		European American I [9]	433.8	448.6	
		African American II [9]	427.4	464.0	
		British (Aberdeen, UK)^*∗*^ [2]	428	459	448
		African American I [9]	434.5	471.0	

Proximal	HSD (mm)	French^*∗*^ [28]			43
		Korean (this study)	43.25	48.50	45.50
		French^*∗*^ [22]			45.6
		Turkish [11]			45.8
		American (Texas, USA)^*∗*^ [23]			46.1
		Pakistani [31]			50.1
	HCO (mm)	Korean (this study)	37.26	38.69	37.88
		Swiss, French (Caucasian) [27]			40.5
		French^*∗*^ [22]			41.0
		Pakistani [31]			41.9
		Turkish [11]			42.7
		American (Texas, USA)^*∗*^ [23]			43
		French^*∗*^ [28]			47.0
	NA2D (deg.)	French^*∗*^ [28]			122.9
		French^*∗*^ [22]			123.1
		American (Texas, USA)^*∗*^ [23]			124.7
		Turkish [11]			128.4
		Swiss, French (Caucasian) [27]			129.2
		Korean (this study)	130.80	129.56	130.27
		Pakistani [31]			130.3

^*∗*^Specific population not mentioned; samples were considered to be from the country of the authors' institute.

HOB: height measured by osteometric board, HSD: sphere diameter fit to head, HCO: head center offset, and NA2D: neck angle on coronal plane.

**Table 4:** Comparison of parameters of the diaphysis among populations.

Measurement		Population	Female	Male	Combined
Group	Abbreviation				
Diaphysis	CL (mm)	Japanese III [51]	254.0	273.5	265.7
		Japanese II [51]	268.5	293.5	280.4
		Korean (this study)	274.47	289.61	281.11
		Japanese I [51]	293.2	311.4	285.3
		North American Indian III [51]	299.3	316.7	308.2
		Inuit [9]	305.5	318.4	312.1
		North American Indian I [9]	302.6	329.6	319.1
		North American Indian II [9]	313.8	327.7	320.9
		African American II [9]	318.4	339.6	329.0
		European American I [9]	322.5	330.6	329.4
		European American II [51]	317.3	343.2	330.2
		African American I [9]	319.6	342.7	332.1
	ST (mm)	African American I [9]	8.4	8.6	8.5
		European American II [51]	8.4	9.3	8.8
		African American II [9]	9.2	9.0	9.1
		Japanese III [51]	9.2	9.1	9.1
		European American I [9]	9.4	9.8	9.7
		Inuit [9]	9.7	11.0	10.3
		Japanese I [51]	9.6	11.6	10.5
		North American Indian II [9]	9.8	11.6	10.7
		North American Indian III [51]	11.0	11.0	11.0
		Japanese II [51]	10.7	12.5	11.6
		North American Indian I [9]	10.9	12.2	11.7
		Korean (this study)	13.82	13.43	13.65

CL: chord length; ST: subtense.

**Table 5:** Comparison of parameters of the distal femur and medullary canal among populations.

Measurement		Population	Female	Male	Combined
Group	Abbreviation				
Distal	DLC (mm)	Korean (this study)	58.39	64.63	60.11
		German^*∗*^ [36]	63.1	69.3	
	WLC (mm)	Japanese^*∗*^ [40]			24.8
		Taiwanese^*∗*^ [38]			25.3
		Korean (this study)	24.05	27.96	25.76
		German^*∗*^ [36]	26.0	30.6	
	DMC (mm)	Korean (this study)	55.25	61.22	57.85
		German^*∗*^ [36]	62.3	69.3	
	WMC (mm)	Korean (this study)	23.46	25.78	24.47
		Taiwanese^*∗*^ [38]			26.7
		Japanese^*∗*^ [40]			30.1
		German^*∗*^ [36]	28.4	32.3	
	DIN (mm)	Korean (this study)	27.16	30.50	28.61
		German^*∗*^ [36]	30.3	32.5	
	WIN (mm)	Taiwanese^*∗*^ [38]			18.2
		German^*∗*^ [36]	19.0	19.3	
		Korean (this study)	18.97	21.66	20.14

Medullary canal	MLWI (mm)	Korean (this study)	9.59	9.6	9.6
		Turkish [11]			10.7
		American (Texas, USA)^*∗*^ [23]			12.3
	APWI (mm)	Korean (this study)	10.97	11.51	11.24
		Turkish [11]			13.7
		American (Texas, USA)^*∗*^ [23]			16.9

^*∗*^Specific population not mentioned; samples were considered to be from the country of the authors' institute.

DLC: depth of lateral condyle, WLC: width of lateral condyle, DMC: depth of medial condyle, WMC: width of medial condyle, DIN: depth of intercondylar notch, WIN: width of intercondylar notch, MLWI: mediolateral width at isthmus, and APWI: anteroposterior width at isthmus.

## References

[1] Trudell M. B. (1999). Anterior femoral curvature revisited: race assessment from the femur. *Journal of Forensic Sciences*.

[2] Bruns W., Bruce M., Prescott G., Maffulli N. (2002). Temporal trends in femoral curvature and length in medieval and modern Scotland. *American Journal of Physical Anthropology*.

[3] De Groote I. (2011). Femoral curvature in Neanderthals and modern humans: a 3D geometric morphometric analysis. *Journal of Human Evolution*.

[4] Gilbert B. M. (1976). Anterior femoral curvature: its probable basis and utility as a criterion of racial assessment. *American Journal of Physical Anthropology*.

[5] Harma A., Karakas H. M. (2007). Determination of sex from the femur in Anatolian Caucasians: a digital radiological study. *Journal of Forensic and Legal Medicine*.

[6] Karakaş H. M., Harma A. (2008). Femoral shaft bowing with age: a digital radiological study of Anatolian Caucasian adults. *Diagnostic and Interventional Radiology*.

[7] Singh S. P., Singh S. (1972). A study of anterior femoral curvature in Indian subjects. *Acta Anatomica*.

[8] Stewart T. D. (1962). Anterior femoral curvature: its utility for race identification. *Human Biology*.

[9] Walensky N. A. (1965). A study of anterior femoral curvature in man. *The Anatomical Record*.

[10] Kim D.-I., Kwak D.-S., Han S.-H. (2013). Sex determination using discriminant analysis of the medial and lateral condyles of the femur in Koreans. *Forensic Science International*.

[11] Atilla B., Oznur A., Caglar O., Tokgözoglu M., Alpaslan M. (2007). Osteometry of the femora in Turkish individuals: a morphometric study in 114 cadaveric femora as an anatomic basis of femoral component design. *Acta Orthopaedica et Traumatologica Turcica*.

[12] Haraguchi K., Sugano N., Nishii T. (2001). Comparison of fit and fill between anatomic stem and straight tapered stem using virtual implantation on the ORTHODOC workstation. *Computer Aided Surgery*.

[13] Hermann K. L., Egund N. (1997). CT measurement of anteversion in the femoral neck: the influence of femur positioning. *Acta Radiologica*.

[14] Ito M., Wakao N., Hida T. (2010). Analysis of hip geometry by clinical CT for the assessment of hip fracture risk in elderly Japanese women. *Bone*.

[15] Kaneuji A., Matsumoto T., Nishino M., Miura T., Sugimori T., Tomita K. (2000). Three-dimensional morphological analysis of the proximal femoral canal, using computer-aided design system, in Japanese patients with osteoarthrosis of the hip. *Journal of Orthopaedic Science*.

[16] Khmelnitskaya E., Mohandas P., Walker P. S., Muirhead-Allwood S. K. (2008). Optimizing for head height, head offset, and canal fit in a set of uncemented stemmed femoral components. *HIP International*.

[17] Kim K. M., Brown J. K., Kim K. J. (2011). Differences in femoral neck geometry associated with age and ethnicity. *Osteoporosis International*.

[18] Laine H.-J., Lehto M. U. K., Moilanen T. (2000). Diversity of proximal femoral medullary canal. *The Journal of Arthroplasty*.

[19] Lausten G. S., Jorgensen F., Boesen J. (1989). Measurement of anteversion of the femoral neck. Ultrasound and computerised tomography compared. *The Journal of Bone and Joint Surgery—British Volume*.

[20] Lecerf G., Fessy M. H., Philippot R. (2009). Femoral offset: anatomical concept, definition, assessment, implications for preoperative templating and hip arthroplasty. *Orthopaedics & Traumatology: Surgery & Research*.

[21] Mahaisavariya B., Sitthiseripratip K., Tongdee T., Bohez E. L. J., Vander Sloten J., Oris P. (2002). Morphological study of the proximal femur: a new method of geometrical assessment using 3-dimensional reverse engineering. *Medical Engineering & Physics*.

[22] Massin P., Geais L., Astoin E., Simondi M., Lavaste F. (2000). The anatomic basis for the concept of lateralized femoral stems: a frontal plane radiographic study of the proximal femur. *The Journal of Arthroplasty*.

[23] Noble P. C., Alexander J. W., Lindahl L. J., Yew D. T., Granberry W. M., Tullos H. S. (1988). The anatomic basis of femoral component design. *Clinical Orthopaedics and Related Research*.

[24] Unnanuntana A., Toogood P., Hart D., Cooperman D., Grant R. E. (2010). Evaluation of proximal femoral geometry using digital photographs. *Journal of Orthopaedic Research*.

[25] Casper D. S., Kim G. K., Parvizi J., Freeman T. A. (2012). Morphology of the proximal femur differs widely with age and sex: relevance to design and selection of femoral prostheses. *Journal of Orthopaedic Research*.

[26] Ferris B. D., Kennedy C., Bhamra M., Muirhead-Allwood W. (1989). Morphology of the femur in proximal femoral fractures. *The Journal of Bone & Joint Surgery—British Volume*.

[27] Husmann O., Rubin P. J., Leyvraz P.-F., De Roguin B., Argenson J.-N. (1997). Three-dimensional morphology of the proximal femur. *The Journal of Arthroplasty*.

[28] Rubin P. J., Leyvraz P. F., Aubaniac J. M., Argenson J. N., Esteve P., de Roguin B. (1992). The morphology of the proximal femur: a three-dimensional radiographic analysis. *The Journal of Bone & Joint Surgery—British Volume*.

[29] Schumann S., Tannast M., Nolte L.-P., Zheng G. (2010). Validation of statistical shape model based reconstruction of the proximal femur—a morphology study. *Medical Engineering and Physics*.

[30] Sugano N., Noble P. C., Kamaric E. (1998). A comparison of alternative methods of measuring femoral anteversion. *Journal of Computer Assisted Tomography*.

[31] Umer M., Sepah Y. J., Khan A., Wazir A., Ahmed M., Jawad M. U. (2010). Morphology of the proximal femur in a Pakistani population. *Journal of Orthopaedic Surgery*.

[32] Cheng X., Li J., Lu Y., Keyak J., Lang T. (2007). Proximal femoral density and geometry measurements by quantitative computed tomography: association with hip fracture. *Bone*.

[33] Khang G., Choi K., Kim C.-S., Yang J. S., Bae T.-S. (2003). A study of Korean femoral geometry. *Clinical Orthopaedics and Related Research*.

[34] Chaichankul C., Tanavalee A., Itiravivong P. (2011). Anthropometric measurements of knee joints in Thai population: correlation to the sizing of current knee prostheses. *The Knee*.

[35] Cheng F. B., Ji X. F., Lai Y. (2009). Three dimensional morphometry of the knee to design the total knee arthroplasty for Chinese population. *The Knee*.

[36] Dargel J., Michael J. W. P., Feiser J., Ivo R., Koebke J. (2011). Human knee joint anatomy revisited: morphometry in the light of sex-specific total knee arthroplasty. *The Journal of Arthroplasty*.

[37] Hitt K., Shurman J. R., Greene K. (2003). Anthropometric measurements of the human knee: correlation to the sizing of current knee arthroplasty systems. *The Journal of Bone & Joint Surgery Series A*.

[38] Ho W.-P., Cheng C.-K., Liau J.-J. (2006). Morphometrical measurements of resected surface of femurs in Chinese knees: correlation to the sizing of current femoral implants. *The Knee*.

[39] Murshed K. A., Çiçekcibaşi A. E., Karabacakoglu A., Şeker M., Ziylan T. (2005). Distal femur morphometry: a gender and bilateral comparative study using magnetic resonance imaging. *Surgical and Radiologic Anatomy*.

[40] Urabe K., Miura H., Kuwano T. (2003). Comparison between the shape of resected femoral sections and femoral prostheses used in total knee arthroplasty in Japanese patients: simulation using three-dimensional computed tomography. *The Journal of Knee Surgery*.

[41] Victor J. (2009). Rotational alignment of the distal femur: a literature review. *Orthopaedics & Traumatology: Surgery & Research*.

[42] Lim H.-C., Bae J.-H., Yoon J.-Y., Kim S.-J., Kim J.-G., Lee J.-M. (2013). Gender differences of the morphology of the distal femur and proximal tibia in a Korean population. *The Knee*.

[43] Poilvache P. L., Insall J. N., Scuderi G. R., Font-Rodriguez D. E. (1996). Rotational landmarks and sizing of the distal femur in total knee arthroplasty. *Clinical Orthopaedics and Related Research*.

[44] Charlton W. P. H., St. John T. A., Ciccotti M. G., Harrison N., Schweitzer M. (2002). Differences in femoral notch anatomy between men and women: a magnetic resonance imaging study. *The American Journal of Sports Medicine*.

[45] Tillman M. D., Smith K. R., Bauer J. A., Cauraugh J. H., Falsetti A. B., Pattishall J. L. (2002). Differences in three intercondylar notch geometry indices between males and females: a cadaver study. *The Knee*.

[46] Kwak D., Han S., Han C. W. (2010). Resected femoral anthropometry for design of the femoral component of the total knee prosthesis in a Korean population. *Anatomy & Cell Biology*.

[47] Kwak D. S., Tao Q. B., Todo M., Jeon I. (2012). Determination of representative dimension parameter values of Korean knee joints for knee joint implant design. *Proceedings of the Institution of Mechanical Engineers, Part H: Journal of Engineering in Medicine*.

[48] Noble P. C., Box G. G., Kamaric E., Fink M. J., Alexander J. W., Tullos H. S. (1995). The effect of aging on the shape of the proximal femur. *Clinical Orthopaedics and Related Research*.

[49] Feik S. A., Thomas C. D. L., Bruns R., Clement J. G. (2000). Regional variations in cortical modeling in the femoral mid-shaft: sex and age differences. *American Journal of Physical Anthropology*.

[50] Martin R. B., Atkinson P. J. (1977). Age and sex-related changes in the structure and strength of the human femoral shaft. *Journal of Biomechanics*.

[51] Shackelford L. L., Trinkaus E. (2002). Late Pleistocene human femoral diaphyseal curvature. *American Journal of Physical Anthropology*.

[52] Kim J. S., Park T. S., Park S. B., Kim S. I. (2000). Measurement of femoral neck anteversion in 3D. Part 2: 3D modelling method. *Medical & Biological Engineering and Computing*.

[53] Seo J.-G., Kim B.-K., Moon Y.-W. (2009). Bony landmarks for determining the mechanical axis of the femur in the sagittal plane during total knee arthroplasty. *Clinics in Orthopedic Surgery*.

